# Nanomedicine-Based Delivery Strategies for Breast Cancer Treatment and Management

**DOI:** 10.3390/ijms23052856

**Published:** 2022-03-05

**Authors:** Priti Tagde, Agnieszka Najda, Kalpana Nagpal, Giriraj T. Kulkarni, Muddaser Shah, Obaid Ullah, Sebastian Balant, Md. Habibur Rahman

**Affiliations:** 1 Amity Institute of Pharmacy, Amity University, Noida 201301, India; tagde_priti@rediffmail.com (P.T.); kalpananagpal@gmail.com (K.N.); 2 Department of Vegetable and Herbal Crops, University of Life Sciences in Lublin, 50A Doświadczalna Street, 20-280 Lublin, Poland; sebastianbalant@o2.pl; 3 Gokaraju Rangaraju College of Pharmacy, Bachupally, Hyderabad 500090, India; gtkulkarni@gmail.com; 4 Natural and Medical Sciences Research Center, University of Nizwa, P.O. Box 33, Birkat Mauz, Nizwa 616, Oman; muddasershah@awkum.edu.pk (M.S.); obaidullah@unizwa.edu.com (O.U.); 5 Department of Global Medical Science, Wonju College of Medicine, Yonsei University, Wonju 26426, Korea

**Keywords:** nanomedicine, drug therapy, breast cancer, targeted delivery, drug resistance, combinational approach

## Abstract

Breast cancer is one of the most common types of cancer among women globally. It is caused by mutations in the estrogen/progesterone receptors and conventional treatment methods are commonly utilized. About 70–80 percent of individuals with the early-stage non-metastatic disease may be cured. Conventional treatment is far less than the optimal ratio, as demonstrated through the high mortality rate of women with this cancer. However, conventional treatment methods like surgery, radiotherapy, and chemotherapy are not as effective as expected and lead to concerns about low bioavailability, low cellular uptake, emerging resistance, and adverse toxicities. A nanomedicine-based approach is a promising alternative for breast cancer treatment. The present era is witnessing rapid advancements in nanomedicine as a platform for investigating novel therapeutic applications and modern intelligent healthcare management strategies. This paper focuses on nanomedicine-based therapeutic interventions that are becoming more widely accepted for improving treatment effectiveness and reducing undesired side effects in breast cancer patients. By evaluating the state-of-the-art tools and taking the challenges involved into consideration, various aspects of the proposed nano-enabled therapeutic approaches have been discussed in this review.

## 1. Introduction

Breast cancer is the most often diagnosed cancer in women and the leading cause of death. Metastasis and tumor recurrence are posing new problems in the management of breast cancer [1]. Over the past few years, breast cancer has risen as one of the most damaging cancers, producing millions of new cases per year [2]. The lobes, lobules (milk producing glands), and bulbs are linked by ducts that lead to the nipple in the centre of a dark area of skin known as the areola. Each breast also contains blood vessels that lead to the small bean-shaped organs, known as lymph nodes, and the vessels that carry lymph. Most of the lymphatic vesselsflow moves toward the axillary and internal mammary lymph nodes, which are located around the breast’s edges in the underarm, above the collarbone, and in the chest. The axillary lymph nodes located underarm are often the major route of regional spread in the metastasis of the primary breast cancer metastasis [3]. When tumor cells infiltrate surrounding normal cells or metastasize (spread) to other areas of the body, the tumor mass becomes malignant. Breast cancer is more often associated with women, although males also have a slight possibility of developing the disease [4]. Breast cancer is treated by a full or partial mastectomy, with radiation after a partial mastectomy or breast-conserving surgery. Complete mastectomy, on the other hand, is said to be linked to long-term survival [5]. Chemotherapy is occasionally used after a mastectomy to ensure that all metastatic cancer cells are removed from the patient’s body [6].

According to global cancer (GLOBOCAN) statistics, female breast cancer has now surpassed lung cancer as the leading cause of global cancer incidence in 2020 with an expected 2.3 million new cases, representing 11.7 percent of all cancer cases and total cancer deaths (6.9%) globally [7]. Breast cancer is most prevalent (>80 per 100,000) in Australia/New Zealand, Western Europe (the world’s highest incidence is in Belgium), Northern America, and Northern Europe, with the lowest rates (40 per 100,000) in Central America, Eastern and Middle Africa, and Southern Central Asia [7].

The American Joint Committee on Cancer (AJCC) is in control of the tumor-node-metastasis (TNM) method for breast cancer staging that regulates how cancer is categorized and conveyed [8]. T, N, and M were used to determine the stage number, the size of the cancer tumor, and whether it has spread to surrounding tissue (T), lymph nodes (N), or progressed beyond the breast to other regions of the body (M). Apart from these, there are five stages of breast cancer, which are numbered as 0, 1, 2, 3, and 4 [9,10] and arementioned in Table 1.

**Figure 1:**
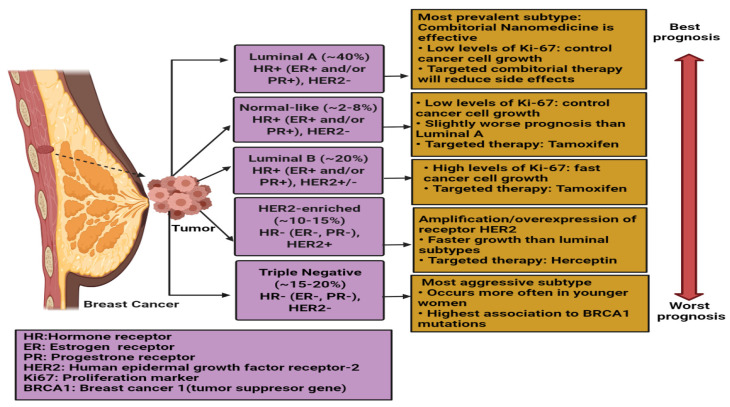
Five Intrinsic or Molecular Subtypes of Breast Cancer with Best Prognosis. Created in BioRender.com.

Patients with breast cancer are frequently given multimodality treatment, which includes standard modalities like surgery, radiation therapy, and medication therapy, as well as optional complementary therapies like acupuncture and diet management. The first two modalities are mostly employed to eliminate primary breast tumors and malignant tissues in the locoregional area and, as the cancer grows and metastasizes, their efficacy declines. Our attention is focused on the final treatment option, pharmacological therapy, which is used to minimizetumor burden and prevent, control, or treat cancer spread. Hormonal therapy, which involves hormones or hormone-like substances, is frequently used in the treatment of breast cancer [18].Recent advances in molecular biology and immunotherapy have increased the inclusion of targeted medicines suited to the unique pathophysiology of several breast cancer subtypes. This technique often includes the use of a small chemical or monoclonal antibody to target a particular molecular pathway (Figure 1 and Table 2), therefore preventing or controlling cancer proliferation, progression, dissemination, and/or treatment resistance.Trastuzumab (herceptin) is the most well-known targeted treatmentup to date. It is a humanized anti-HER2 monoclonal antibody [18]. At the moment, adjuvant therapy treatment is mostly determined by the intrinsic subtype of breast cancer. The conventional drug therapy options are summarized in Table 2.

Abemaciclib, palbociclib, and ribociclib are three novel compounds that have been shown to be effective against breast cancer metastasis in the past five years. They inhibit CDK 4/6 and serine/threonine kinases that are elevated in a variety of TCs [19]. These drugs are recommended to treat metastatic breast cancer in hormone receptor–positive (HR+) and HER2-negative (HER2−) patients and are considered a successful innovation in the cancer pharmaceutical industry due to their oral usage and good pharmacological responses. The National Cancer Institute offers 63 commercially available drugs for breast cancer therapy that have been approved by the United States Food and Drug Administration for use alone or in combination [19]. The most significant ones are shown in Table 3. Few of these are dependent on the existence of hormone receptors (ER and PR) and/or the expression of the HER2 protein. With the exception of CDK4/6 inhibitors, the majority of current drugs still focus on nonselective modes of action, such as cell cytotoxicity and cell division inhibition, resulting in poor patient quality of life.

Despite all efforts, few molecules with novel mechanisms of action have been produced for anticancer drug development programs. Thus, investigations into the fundamental physiology of cancer cells from various viewpoints may provide novel insights and treatments.

As there are no drugs available that inhibit cell extravasation, examining the cell environment and its communication with the micro-environment and other cells may be a potential method for anti-metastatic drug development. Numerous preclinical and clinical investigations have identified many novel targets, the majority of which are promising for treating or preventing metastasis. Efforts to build high-throughput models for testing these targets, as well as libraries of novel and recognized molecules, are critical to avoiding the time-consuming process of developing drug treatments. Furthermore, nanomedicine has also been envisioned to reduce the quantity and frequency of doses while keeping a similar pharmacological profile and adverse effects (Table 4). As well, the ability of nanocarriers to disperse in confined tissues and for nanomedicine to target breast cancer cells overexpressing HER2 and target TNBC and breast cancer stem cells (BCSCs) have been envisioned. 

This review highlights the most recent and relevant published results on nanomedicine-based drug delivery alone or in combinatorial therapy, which are promising approaches utilized to combat the aforementioned limitations associated with most anticancer drugs and involves the concurrent administration of two or more anticancer agents or phytoconstituents with different modes of action to overcome multidrug resistance. These approaches are increasingly acceptable to improve therapeutic efficacy and reduce unwanted effects associated with an incorporated anticancer payload in breast cancer.

## 2. Nanomedicine: Evolving Demands for Breast Cancer Treatment

Nanomedicine in the medical or pharmaceutical field appears to be a new trend and has been proposed to minimize dose quantity and frequency while retaining a similar pharmacological profile and fewer side effects [40], as well as nanosystems that enable them to push through biological barriers like the blood-brain barrier (BBB) [40]. Among these, nano-lipid carriers for cancer therapy will lead to improved features such as higher drug loading capability, strong compatibility, scaling up viability, and regulated drug release [41]. Nanoparticles are commonly used to synthesize and prepare anti-infective, anticancer, and anti-inflammatory medicines [42]. The nanoparticles vary in size from 1–100nm. The nanoparticles combine in a multi-layered fashion, and the coating aids in resolving issues such as solubility, durability, and specificity. Using a nanoparticle-based technique, problems correlated with macromolecules such as cell toxicity, lacking specificity, cellular absorption, and the high dose may be avoided. In contrast, concerns linked to multidrug resistance (MDR) and *P*-glycoprotein (*P*-*gp*) efflux may also be changed [43].

The nanoparticle’s wavelength, which is usually less than 100 nm, will provide inherent stealth. Due to the drug’s lipophilic nature, it is simple to entrap and formulate into nanoparticles [44]. The conjugation of the parent first-line chemotherapeutic agent and the flavonoid with a lipid improves the drug’s lipophilicity, which aids in enhancing the entrapment efficiency when forming nanodroplets. The nano-emulsion system’s buoyant charge aids in communicating with the negative charge on the surface of cancer cells. This aids in the successful transmission of nanoparticles to the cancer cell surfaces [45]. There have been no records of the nanoparticles having any negative or harmful results since being delivered in 2D person and in vivo [46]. These are supported by an evidential study demonstrating that toxicity-related conditions are rare and have little impact on the brain, heart, lungs, or kidney. According to one study, nanoparticle aggregation in body parts such as the liver and spleen has been seen [47]. Nanomedicine is being used in many of the formulation approaches mentioned above; there are two commonly acknowledged approaches: (a) top-down method and (b) bottom-up technique. In the top-down method, the required non-material is made by using external, macroscopic raw materials. The processing of these macroscopic materials is well-controlled from the outside. Etching, ball-milling, homogenization, and the application of strong plastic deformations are all examples of this kind of method [48]. In the bottom-up method, the raw material is pre-miniaturized (at the molecular/atomic level) and then either allowed to self-assemble into nanomaterials or additional catalysts are included to aid assembly. Other molecules of interest may be introduced into the nanomaterial to create composite nanoproducts during the construction process. However, the end output must be “stabilized” by some external means in each of these ways. Otherwise, these fundamentally unstable nanoproducts tend to agglomerate while stored [48] and it is having a positive impact on the pharmaceutical healthcare industry.

**Figure 2:**
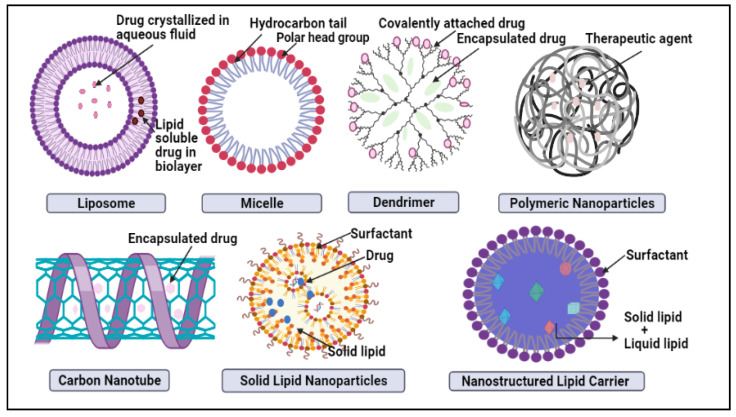
Schematic representation of novel nano-drug delivery approaches in breast cancer treatment.

In nano-delivery, the antibody can coat the nanocarrier surface for targeted distribution to HER2 cells. There have been many recent studies on HER2-targeted nanomedicine; although the HER2 antibody is the most often used targeting moiety for HER2 malignancy, other HER2 targeting ligands have been studied as well. For example, Ding et al. used a trastuzumab-mimetic peptide with promising results, indicating that this might reduce the antibody’s immunogenicity, production costs, and technical effort [49]. The nanoparticles developed by Day et al. [50] and Cai et al. [51] are both decorated with trastuzumab coating for targeted photoablation and radiation therapy. The HER2 protein, which fuels this kind of breast cancer, might be targeted to slow it down potentially. Monoclonal antibodies are synthesized proteins that attack and inhibit the proliferation of HER2 cells. These medications may be prescribed alone or in conjunction with chemotherapy by doctors. Breast cancer can also be treated with an antibody–drug combination, which delivers pinpoint accuracy chemotherapy to the cancer cells [52]. There are various nanocarriers like liposomes, carbon nanotubes, micelles, dendrimers, metallic nanoparticles (NPs), nanocrystals, polymeric NPs, and lipid-based nanocarriers, such as SLN and NLC, which are examples of some of the nanocarrier systems in Figure 2 and their advantages and disvantages illustrated in Table 5 [53]. The benefit of nanocarriers is their compact scale, which enables them to push through biological barriers like the blood-brain barrier (BBB) [54]. As well, the nanocombinatorial approaches have shown promising results to treat breast cancer [55].

### 2.1. Liposomes

Liposomes are spherical entities that are self-contained and made up of one or more concentric curved bilayer membranes as well as cholesterol. Liposomes are made up of components that are comparable to those found in the cell membrane [60]. Since one side of a phospholipid molecule is hydrophobic and the other end is hydrophilic, when coupled with water, they instantly form a spherical particle [61]. Liposomes have excellent possibilities for medication and cell delivery, owing to the amphiphilic nature of the lipids. Liposomes are necessary for regulated medication and gene delivery to the target region, as well as cell proliferation inside the pores or surrounding tissue. In vivo, the matrix phase should biodegrade at a controlled pace and elicit a low immunological and inflammatory response [62].

Insufficient drug concentrations due to poor absorption, excessive metabolism and elimination, low water solubility, and large plasma level variations due to variable bioavailability after oral administration were usually accompanied by unsatisfactory in vivo results [63]. The development of appropriate drug carrier systems is a possible solution for tackling these issues. When combined with trastuzumab, liposomal anthracyclines are effective in both progressed and early breast cancer [64]. In patients with early and HER2-over-expressing breast cancer, the use of a combination of liposomal anthracyclines and trastuzumab is of particular interest. This is the group that is most likely to benefit from anthracycline treatment [65].

Chowdhury, N. et al. [66] developed an aptamer (A6 and GFP)-labeled liposomal nanoparticle delivery system that retains and distributes doxorubicin to HER-2+ breast cancer cells. As well, it shows a significant increase in the uptake of the aptamer-labeled liposomes—by more than 60%—into both MCF-7 and SKBR-3 cells compared to non-aptamer-labeled nanoparticles and an improved uptake in HER-2 positive cells than HER-2 negative cells. Formulation shows ≈ 1.79-fold increase in the uptake of DOX in the HER-2+ cells compared to the HER-2- cells. This preliminary study indicates that aptamer-labeled nanoparticles, among several batches, showed the highest uptake as well as the targeted delivery of doxorubicin into HER-2+ breast cancer cells. Thus, an aptamer targeted approach results in a substantial reduction in the dose of DOX and improves the therapeutic benefits by promoting the target specificity. Commercialized/marketed cancer nanomedicine liposomal formulation clinical trials are mentioned in Table 6.

Doxil^®^, Janssen Products, Titusville, NJ, USA is a pegylated liposomal DOX HCl formulation that reduces systemic toxicity while maintaining DOX antitumor properties, significantly reducing tumor growth rates, and enhancing survival rates [67]. Doxil helps alleviate the cumulative dosage limitation and allows for lower risk and longer DOX therapy, thereby significantly increasing the drug’s flexibility. In clinical trials for advanced breast cancer treatment, Doxil has been coupled with a wide range of other chemotherapeutic agents (e.g., cyclophosphamide and 5-fluorouracil, cisplatin and infusional fluorouracil, cyclophosphamide followed by paclitaxel, cyclophosphamide accompanied by paclitaxel [68].

Lipodox^®^ is a DOX HClliposome injection that is generic. Monotherapy is used for the treatment of metastatic breast cancer with a high risk of cardiac complications and can reduce the danger of infusion responses. For this process, the drug is given intravenously at a dosage of 50 mg/m^2^ at a rate of 1 mg/min at first and is administered once every four weeks for as long as the patient reacts well and tolerates the therapy [69]. When combined with Lapatinib, dHER2+AS15 ASCI was assessed by dose-limiting toxicities. There were two rounds of intramuscular injections every two weeks, and, between immunization rounds, there was a four-week break. For each dose of 500g of dHER2 + AS15 ASCI, two sterile glass vials were provided: one vial will contain this same lyophilized preparation containing 500 μg of recombinant dHER2 antigen, especially in combination with the immunostimulant, and the other vial will contain the dried preparation containing 500 μg of recombinant dHER2 antigen combined with the immunostimulant. The final dHER2 + AS15 ASCI for administration is obtained by reconstitution of the lyophilized product with adjunct diluents. The dose of dHER2 + AS15 ASCI is 0.5 mL [70].

Lipoplatin, an effective liposomal formulation of cisplatin, is an intriguing medication in breast cancer, especially in HER 2-negative and triple-negative patients—although its efficacy has to be confirmed [71].

EndoTAG-1/MediGene is another Liposomal paclitaxel formulation successful in TNBC with a weekly dosage of EndoTAG-1 22 mg/m^2^ + Paclitaxel 70 mg/m^2^ and a 4-month progression-free survival (PFS) rate [72]. The DPX-0907 vaccine is safe and well-tolerated. This may increase patient survival by inducing efficient anti-tumor immunity [73]. LEM-ETU is a liposomal formulation containing Mitoxantrone, an anticancer drug given intravenously every 21 days until disease progression or toxicity occurs, which requires treatment discontinuation [74]. Table 6 illustrates the various liposomal formulations of cancer nanomedicines, including their brand name, the clinical trial phase, and company name.

Dual-targeting liposome modified by glutamic hexapeptide and folic acid for bone metastatic breast cancer is also an effective formulation [81]. Liang, Z. et al. [82] illustrated targeted delivery of siRNA via a polypeptide-modified liposome for the treatment of gp96 over-expressed breast cancer. There are several metallic liposomalformulation, such as Ru(III) complexes [83,84] with nucleolipids (AziRu), that the authors were able to create with differently decorated anticancer nanosystems, which were very effective against human BCC. In the current landscape of Ru-based candidate drugs, the authorshave demonstrated that AziRu, when inserted into a nucleolipidic structure and ad hoc nano-delivered via the positively charged lipid DOTAP, can effectively inhibit BCC proliferation in vivo while being well-tolerated, which is a critical property for anticancer drug candidates in preclinical studies to progress to the clinical stage. Thus, they demonstrated the safety and effectiveness of HoThyRu/DOTAP cationic Ru-based nanosystems in a mouse xenograft model of BC. 

### 2.2. Dendrimers

Dendrimers are promising drug delivery devices that can solve the shortcomings of currently approved anticancer medications [85]. They can overcome drug resistance, decrease drug toxicity, and increase drug solubility and bioavailability [85]. Anticancer medicines have been loaded into micelles and dendrimers, resulting in selective drug delivery, sustained drug release, improved cellular uptake, decreased adverse side effects, and enhanced anticancer action in vitro and in vivo. Dendrimers are typically used to accomplish successful drug targeting to tumor tissues by covalently binding unique targeting moieties such as folic acid, antibody, sugar epidermal growth factor, and biotin [85]. Chemotherapeutic agents and theranostic chemotherapy applications have been successfully administered using these nanocarriers [86].

Poly-lysine, polypropylene imine (PPI), phosphorus, and carbosilane dendrimers are other forms of dendrimers used in biomedical applications, especially in oncology [87]. Polylysine (PLL) is an amphiphilic dendrimer with a branched structure made up of Penta-functional central molecules that are made up of positively charged essential amino acids, including lysine-amino-alanine, and are a fascinating new class of molecules because of their small size and natural components, which allow them to be internalized more readily than synthetic molecules [88]. The therapeutic efficacy of dendrimers and micelles for breast cancer treatment was reviewed by Sibusiso Alven et al. [89]. The latter demonstrated how they could resolve drug resistance, decrease drug toxicity, and increase drug solubility and bioavailability. Anticancer medications have been loaded into micelles and dendrimers, resulting in selective drug delivery, sustained drug release, improved cellular absorption, decreased adverse side effects, and enhanced anticancer action in vitro and in vivo [90]. The biological impact of dendrimers and micelles loaded with various recognized anticancer agents on breast cancer in vitro and in vivo are reported in their studies. Dendrimeric formulation under clinical trial is illustrated in Table 7.

Although the larger G5 PEG1100 Dendrimer showed firm tumor and retention, drug release was poor, which restricted its anticancer effect. The smallest G4 PEG570 dendrimer was substantially effective in DOX release induced by cathepsin, but its systemic exposure and tumor uptake were minimal. Drug release kinetics, tumor absorption, systemic exposure, and retention were all improved with the intermediate-sized dendrimer. These results showed that the therapeutic effectiveness of dendrimer formulations is influenced by the polyethylene glycol (PEG) molecular weight and dendrimer scale [91].

In another study, dendrimer-loaded trastuzumab was conjugated to neratinib. The in vitro viability of SKBR-3 cells after 48 h for neratinib, neratinib-conjugated-dendrimers, and neratinib-loaded-dendrimers-trastuzumab was 40%, 36%, and 33%, respectively. Trastuzumab’s affinity for HER2 receptors expressed in SKBR-3 cells enhanced the internalization of the formulation through receptor-mediated endocytosis [92].

### 2.3. Micelles

Micelles are colloidal particles around 5–100 nm that are currently under investigation as carriers for hydrophobic drugs in anticancer therapy and have excellent tumor-targeted delivery properties, making them a feasible drug delivery mechanism with high translational potential [89].

NC-6300, NK911, and NC-6004 are micelle products engineered to deliver epirubicin, DOX, and cisplatin, respectively [93]. The antitumor efficacy of NC-6300 was shown in a phase I clinical trial in 2013 with a substantial reduction in cardiac toxicity, suggesting that the formulation is safe and tolerable [94]. DOX is mechanically encapsulated in the hydrophobic center of the micelles by noncovalent bonds [95]. SP1049C comprises a non-ionic Pluronic block copolymer mixture (1:8 *w*/*w* ratio) of Pluronic L61 and F127, which was shown to be more effective than doxorubicin against several tumor cell lines in vitro [96]. Compared to free doxorubicin in preclinical in vivo studies, SP1049C showed superior antitumor activity, efficacy, and an increased area under the curve (AUC) in tumor tissue in multiple animal tumor models of doxorubicin-resistant tumors and had identical AUC and Cmax in the liver, kidney, breast, lung, and plasma [97].

Sun Y. et al. [98] developed PAA-g-PEG graft micelles for high doxorubicin loading for specific target antitumor activity against mouse breast carcinoma for TNBC and discovered that articulation of DOX in the micelles, which enhanced the bioavailability of the drugs through passive targeting of the tumor and significantly reduced organ failure owing to wild tumor cell growth and metastasis and depressed the toxicity of DOX on the heart and other organs. Using a novel fluorescent cancer cell model, authors were able to demonstrate enhanced sensitivity of cancer stem cells to paclitaxel using poly[(D,L-lactide-co-glycolide)-co-PEG](PLGA-co-PEG) micelles of paclitaxel with CD44 surface markers [99]. Genexol-PM [100] is a new cremophor EL-free polymeric micelle formulation of paclitaxel. This single arm, multicenter phase II research was aimed to examine the effectiveness and safety of Genexol-PM in patients with histologically proven metastatic breast cancer (MBC). Forty-one women received Genexol-PM by intravenous infusion at 300 mg/m^2^ over 3 h every 3 weeks without premedication until disease progression or intolerability. In Table 8 we mentioned the various cancer nanomedicine micelle formulations with status of clinical trial phase.

Taurin et al. [104] have developed a second-generation curcumin derivative, 3,5-bis(3,4,5-trimethoxybenzylidene)-1-methylpiperidine-4-one (RL71), that shows potent in vitro cytotoxicity. RL71 is hydrophobic with poor bioavailability, which limits its clinical development so authors have designed styrene-co-maleic acid (SMA) micelles encapsulating 5%, 10%, or 15% RL71 by weight/weight ratio to improve its solubility and pharmacokinetic profile.

In another study, authors created a nanocarrier to deliver a chemotherapeutic drug specifically to the TNBC. To form micelles for the encapsulation of docetaxel, d—tocopheryl polyethylene glycol succinate (vitamin E TPGS, or simply TPGS) was employed. Vitamin E TPGS has both a lipophilic alkyl tail and a hydrophilic polar head, which results in micelle formation above the CMC of 0.02 wt%. Additionally, TPGS micelles have a high surface area that may change for ligand conjugation to deliver specific drugs. The fibroblast cells (NIH3T3), HER2 overexpressed breast cancer cells (SK-BR-3), ER and PR overexpressed breast cancer cells (MCF7), and TNBC cells of high, moderate, and low EGFR expression (MDA MB 468, MDA MB 231 and HCC38) were employed to access in vitro cellular uptake of the coumarin 6-loaded TPGS micelles and the cytotoxicity of docetaxel formulated in the micelles. The high IC_50_ value, which is the drug concentration needed to kill 50% of the cells in a designated period, such as 24 h, obtained from Taxotere^®^ showed that the TNBC cells are indeed more resistant to the free drug than the positive breast cancer cells. However, the therapeutic effects of docetaxel have been significantlyenhanced by the formulation of Cetuximab conjugated TPGS micelles, which demonstrated 205.6- and 223.8-fold higher efficiency than Taxotere^®^ for the MDA MB 468 and MDA MB 231 cell lines, respectively [105].

### 2.4. Carbon Nanotubes

Carbon nanotubes are cylinders made up of one or more co-axial graphite layers with a diameter in the nanometer range that serve as instructive examples of nanomaterials with Janus-like properties [106]. Their structure can be divided into single-walled carbon nanotubes with a single cylindrical carbon wall and multi-walled carbon nanotubes with multiple wall cylinders [106]. They can offer a promising approach to gene and drug delivery for cancer therapy due to their unique electronic, thermal, and structural characteristics. Due to their thermal conductivity and optical properties, carbon nanotubes are the right candidate for killing cancer cells via local hyperthermia [107].

Carbon nanotube materials may be used as instruments for targeted and regulated drug distribution and release, contrast agents for diagnosing and identifying breast tumors, and biosensors [108].Fullerenes, carbon nanotubes, and graphene have been shown to have desirable properties for the carriage, targeted, and regulated distribution of chemotherapeutical drugs such as Taxol (paclitaxel), docetaxel (DTX), doxorubicin (DOX), and others, according to recent nanomedicine reports [109,110,111].

Nadrajan Jawahar et al. [112] developed a folic acid-conjugated raloxifene hydrochloride carbon nanotube for targeting breast cancer cells, demonstrating that the surface of the CNTs was functionalized by folic acid (FA), allowing the medicine to be delivered selectively to the cancer cells’ target sites. In vitro, drug release studies revealed that the system’s pH influenced drug release. The effectiveness of FAs physically attached to CNTs with affectivity produces apoptosis in the cancer cell line with an IC50 value of 43.57305 g/mL, according to a cytotoxicity investigation. When compared to the pure medication and the RLX-CNT formulation, the fluorescence imaging investigation revealed that the RLX had increased cellular internalization.

By using the plasma-enhanced chemical by vapor deposition (PECVD) process, Akinoglu, E. M. et al. [113] determined that a multi-walled carbon nanotube-based scaffold has a good shape for cell development and offers a biocompatible environment for human MDA-MB-231 cell lines. The current findings revealed improved cell adherence to the scaffold and displayed excellent biomimetic features and physiological adaptability, suggesting that they may be utilized to research BrCa cell line metastasis in vitro.

### 2.5. Polymeric Nanoparticles

Polymeric nanoparticles (PNPs) are submicron-sized structures made up of several biodegradable (e.g., albumin, chitosan, and alginate) and non-biodegradable (e.g., albumin, chitosan, and alginate) polymers [114]. Therapeutic agents may be encapsulated, covalently bound, or adsorbed to nanocarriers in this manner [114]. These strategies can quickly solve drug solubility problems, which are significant since a substantial percentage of potential drug candidates discovered by high-throughput screening programs are water-insoluble [115]. On the other hand, polymeric nanoparticles are distinguished from drug nanosuspensions, which are sub-micron colloidal dispersions of pure drug particles stabilized by surfactants [116]. Polymeric nanoparticles may also be tailored to individual cells and sites in the body due to their small size and the ability to functionalize their surface with polymers and suitable ligands. As a result, polymeric nanoparticles could solve drug stability problems and reduce drug-induced side effects [117]. Several PNPs were developed and used to treat cancer, especially in the distribution of anti-cancer drugs [118]. Furthermore, cancer nanomedicine under clinical trial is illustrated in Table 9.

Ikmeet Kaur Grewal et al. [119] have reported a summary of recent advances in polymeric nanoparticles for breast cancer care and patents with clinical trial studies based on the recently published Web Of Science data.

### 2.6. Solid Lipid Nanoparticles

Lipid-based nanoparticles, including solid lipid nanoparticles (SLNs) and nanostructured lipid carriers (NLCs), will hopefully overcome the current drawbacks presented by liposomes and polymeric nanoparticles [124]. Lipid nanoparticles show the possibility of discovering new therapeutics due to their unusual size-dependent properties [125]. The ability to integrate drugs into nanocarriers introduces a modern drug delivery prototype that can be used for secondary and tertiary drug targeting [126]. As a result, SLNs hold a lot of promise for achieving the objective of controlled and site-specific drug distribution, and they’ve gotten a lot of attention from scientists [127]. SLNs consist of a solid lipid matrix that is in solid state at both room and body temperatures and are made in the same way as an oil-in-water (*o*/*w*) emulsion, except that the oil phase is substituted by a solid lipid or a mixture of solid lipids. Solid lipids in SLNs include long-chain fatty acids, fatty acid esters, and waxes. The particle size (PS) of SLNs typically varies from 80 to 1000 nm [127]. Since these small particles may not re-crystallize throughout the production process, producing SLNs with a mean PS of less than 80 nm is challenging. The SLNs dispersion may be utilized directly as a nanosuspension or may be integrated into solid dosage forms, such as tablets and pellets, by granulating the dispersion. Alternatively, a spray-drying or lyophilization procedure might be effective in order to transform an aqueous SLN dispersion into a dry product and this will improve the SLN’s long-term stability, and it can be reassembled with water to make a nanosuspension as needed [127]. Table 10 [128] lists the substances usually utilized in the manufacture of SLN.

**Figure 3:**
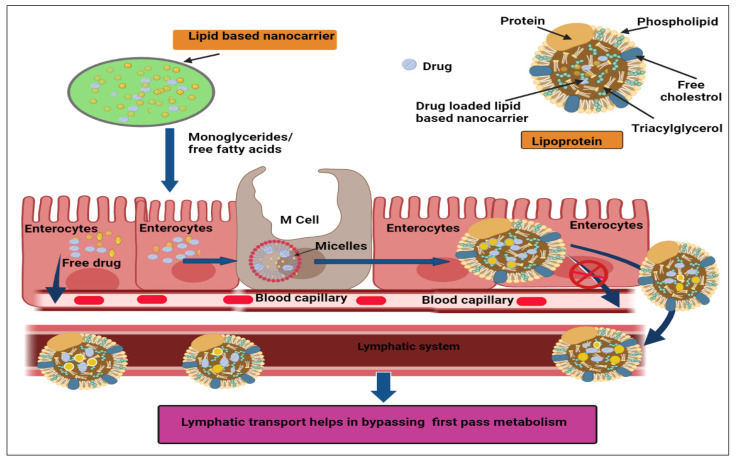
Absorption mechanism of lipid based nanocarriers through lymphatic transport system.

One of the most important factors in breast cancer mortality is metastasis, and the most common sites for metastasis are local lymph nodes [129]. As seen in Figure 3, the rate of tumor cell migration to lymph nodes increases, resulting in the incorporation of lymph vessels, which systemically spread the cancer cells through the lymphatic route. This route can provide new possibilities for delivery of anticancer drugs to overcome first pass metabolism of the drug and can serve as a bypass route. Anticancer drugs, encapsulated in advanced lipid-based nanocarriers such as SLNs and NLCs, are better candidates for lymphatic drug delivery. Lipid digestion occurs in the intestine by m-cells and then they are converted into micelles and transformed into lipoproteins after cholesterol and phospholipid aggregation. Finally, lipoproteins by pass first-pass metabolism and are sent to the lymphatic system. Henceforth, the systemic toxicity profile associated with anticancer drugs can be avoided and breast cancer patients would benefit from such a strategy, which would be safer than conventional chemotherapy.

Other formulations, such as dimethyl sulfoxide solubilization and Cremophor EL vehicles, were linked to paclitaxel-SLN action toward MCF-7 drug-resistant and drug-sensitive cells (commercial formulation). The cytotoxicity of SLNs with paclitaxel was determined to be concentration-dependent [128]. Pindiprolu et al. [129] generated niclosamide-loaded SLNs that increased cellular uptake and chemotherapeutic efficacy in TNBC. Another study, such as their use of curcumin carriers against the breast cancer cell line MDA-MB-231, has also backed up the efficacy of SLNs. When curcumin was injected into the SLNs, the findings revealed a significant improvement in the cells drug absorption capability, and curcumin–SLN also caused a greater decrease in cell viability and a rise in apoptotic cells as compared to curcumin diluted in dimethyl sulfoxide [130]. Another study was on the usage of resveratrol-loaded SLNs to treat human breast cancer cells, which was also a source of concern and investigators discovered that resveratrol SLNs are more efficient at inhibiting cell proliferation than free resveratrol in this case. They also had a much stronger inhibitory effect on cell invasion and migration, implying that resveratrol–SLN may be a promising BreC drug [131]. The history and background of SLNs are very short as the findings were lacking in clinical studies for breast cancer treatment [131]. In Table 10 we have illustrated various solid lipids and surfactants and co-surfactants used in the formulation of SLNs, and in Table 11 we mentioned the formulation of SLNs with different methods by different researchers for breast cancer treatment.

### 2.7. Nanostructured Lipid Carriers (NLC)

NLCs are the second generation of lipid-based nanocarriers formed from a mixture of solid and liquid lipids and have an unstructured matrix due to the different moieties of the constituents of NLCs [144]. NLCs were designed in order to overcome the SLNs’ limitations. NLCs have a higher drug loading capacity because of their imperfect crystal structure and could avoid drug expulsion by avoiding lipid crystallization during the manufacturing and storage periods. Due to the presence of liquid lipids in NLCs formulation, expulsion of the loaded drug after formulation and during the storage period is minimized. NLCs can also increase drug solubility in lipid matrices and they can show more controllable release profiles in comparison to SLNs [145]. Although NLCs are solid in nature, even in body temperature, they have a lower melting point than SLNs and, due to their unstructured nature and imperfection in their crystalline behaviors, provide more space for drug dissolution and payload in the liquid part of the NLC [146].

By replacing liquid lipid for a component of pure solid lipid, the strategies to keep defects in the lipid, even after lengthy storage, alleviate the issues associated with standard SLNs [147]. When a little amount of liquid lipid/oil was added to a solid lipid matrix, the crystal lattice structures were less ordered [148].

Liquid lipids contributed in widening the loading capabilities of lipid nanocarriers by increasing the number of defects wherein amorphous drug clusters might fit. As a consequence, this dual lipid framework may not only be able to accommodate higher drug loads, but it may also be able to reduce the drug’s expulsion from the lipid during storage [149].

Mingzhen Lin et al. [150] formulated a folic acid-loaded curcumin nanostructured lipid carrier using a solvent diffusion approach. The results revealed that FA-CUR-NLCs were efficient in selective delivery to cancer cells overexpressing FA receptors (FRs). CUR is also delivered to breast cancer cells via FA-CUR-NLCs, boosting anti-tumor action. As a consequence, FA-CUR-NLCs might be a more effective nanomedicine for tumor therapy. In Table 12 we have illustrated various solid lipids, liquid lipids, surfactants, and co-surfactants used in the formulation of SLNs, and in Table 13 we mentioned the formulation of NLCs with different methods by different researchers for breast cancer treatment.

## 3. Combinatorial Nanocarrier-Based Drug Delivery for Anti-Tumor Agent Amalgamation in Breast Cancer

In recent years, nanocarrier-based drug delivery has risen, with significant socioeconomic implications in various disciplines [158]. Furthermore, due to the nanocarrier’s capacity to deliver inside limited tissue, reducing dose quantity and frequency has been recommended while maintaining a similar pharmacological profile and fewer adverse effects [159]. Despite advances in surgical procedures and therapy regimes, patients with breast cancer have a poor prognosis. Henceforth, to improve the quality of life of breast cancer patients, cancer treatment strategies have shifted toward a nanomedicine drug delivery with the combinatorial approach to mitigate the toxicological issues associated with monotherapy [160]. Combining a synthetic chemotherapeutic agent with another synthetic or herbal bioactive molecule with anticancer activity has shown promising results in treating various cancers [161]. This amalgamation of a synthetic chemotherapeutic agent with an herbal anticancer bioactive molecule ameliorates the efficacy of monotherapy either synergistically or in an additive manner. Several such combinations have been reported to attenuate the toxicity associated with high-dose monotherapy [46]. These combinations have shown therapeutic benefits at a lower dose of the synthetic chemotherapeutic agent, which can easily be calculated using the combination index [162].

Boroujeni et al. [163] developed and manufactured curcumin-loaded folate-modified-chitosan-NPs with targeting capabilities by using a self-assembling approach. Curcumin release from folic acid chitosan NPs was finally demonstrated to be influenced by the concentration of the release medium, with a drop in pH from 7.4 to 5.0 speeding up the process. Curcumin-loaded NPs have been demonstrated to have an excellent potential for application in breast cancer treatment.

**Figure 4:**
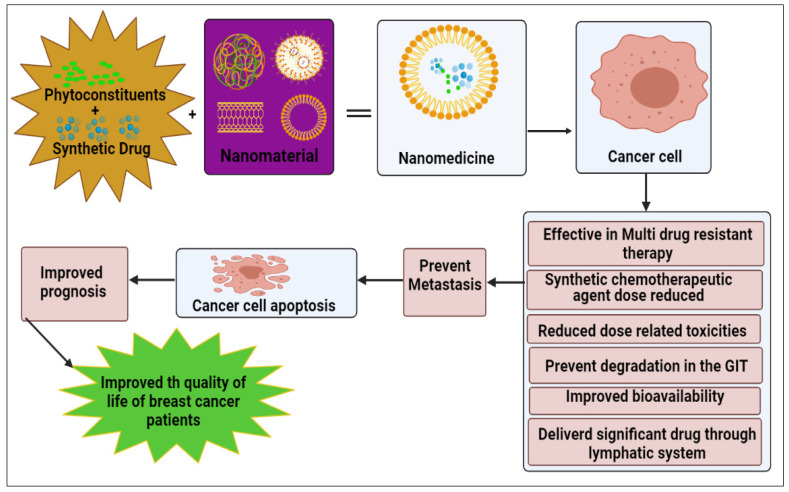
Action of nanomedicine combinatorial approach on breast cancer cells and effectiveness.

Day, C. M. et al. [164] additionally conjugated N-desmethyl tamoxifen and a Zinc (II) phthalocyanine moiety with the non-toxic amphiphilic spacer TEG. The novel conjugate was discovered to have a high affinity for ERs, thereby allowing it to perform both BC photodynamic and hormone treatment. The resultant conjugation has good biocompatibility and it showed an excellent cytotoxicity profile witha50% cancer cell killing impact that had powerful cancer apoptotic capabilities on the MCF-7 cell line. In Table 14 we mentioned the various combinatorial formulations by using different nanocarrier approaches with different methods for breast cancer treatment. The pictorial representation of the action of a nanomedicine combinatorial approach and its action on breast cancer cells is given in Figure 4.

## 4. Perspective &Limitations

In recent years, significant progress has been made in applying nanomedicine for breast cancer treatment. For example, several nanocarriers for breast cancer treatment can now be manufactured in large quantities for commercialization with high stability, bioavailability, and therapeutic effectiveness. Many of the nanocarriers are capable of reducing the dose and toxicities associated with the anticancer drugs. Some of the carriers can be used to specifically target breast cancer tissues. A majority of the excipients used in the nanocarriers are biodegradable and, hence, are environmentally friendly. The nanocarriers need to be covalently linked to the targeting moieties for tissue specific targeting and in combination with synthetic chemotherapeutic agents with an herbal anticancer bioactive molecule, which can overcome the problems of drug resistance and cancer stem cells.

The limitations are also associated, for example, many of the nanocarriers are reported to be toxic to the tissues, can trigger the immune system, and the most severe problem is the nanoformulations increasing the complexity. For example, active targeting is used in the majority of current nanotherapeutic methods for breast cancer therapy. While this is theoretically better than passive targeting, adding targeting moieties to a formulation adds complexity, leading to more significant toxicity and immunogenicity risks, higher production costs, possible up-scalability, and good manufacturing practice problems. This problem may also be seen in multidrug nanoformulations.

## 5. Conclusions

Nanomedicine is a novel treatment alternative for breast cancer that may lower the toxicity and chemoresistance of conventional therapies while also increasing the drug’s anticancer efficiency. This research addressed the promise of nanotherapeutics for breast cancer treatment, the obstacles of anticancer therapy, and future perspectives on the pharmacological significance and anticancer efficiency of nanocarriers. Lipid-based nanocarriers had one of the most promising outcomes of all nanocarriers, although a combination of a synthetic chemotherapeutic agents with an herbal anticancer bioactive molecule can overcome the problems of drug resistance and cancer stem cells. To improve the lives of breast cancer patients, researchers will have to focus on lipid-based nanocarriers utilizing a combinatorial method in the coming decades.

## Figures and Tables

**Table 1:** Various stages of breast cancer along with indication and treatment approach.

Stage	Indication	Treatment Approach
Stage 0	Non-invasive breast cancers, such as ductal carcinoma in situ (DCIS) [11]. There is no sign of cancer cells or non-cancerous aberrant cells breaking out of the breast region where they began [12].	
Stage I	Invasive breast cancers. The cancer either hasn’t spread beyond the breast or has spread in a very small amount to a lymph node [13].	
Stage IA	Cancer has progressed to the fatty tissue of the breast. The tumor is no bigger than a shelled peanut.	Breast-conserving surgery or mastectomy may be used to treat these cancers. A sentinel lymph node biopsy (SLNB) or an axillary lymph node dissection will be required to examine the adjacent lymph nodes (ALND). Hormonal therapy is prescribed for people with hormone-receptor-positive cancer. Immunotherapy may be recommended before and after surgery if the cancer is triple-negative. Targeted therapy, such as trastuzumab (Herceptin), either alone or alongside chemotherapy recommended for HER2 positive [13].
Stage IB	Cancer cells have been discovered in a few lymph nodes, but only in trace levels.	
Stage II	Cancer has grown or spreads [14].	
Stage IIA	Indicates that the breast tumor, if present, is still tiny. It’s possible that there’s no cancer in the lymph nodes or that it’s spread to three or more.	Some systemic therapies may benefit in stage II and are given before surgery (neoadjuvant therapy), whereas others are given after (adjuvant therapy). The drugs utilized will be chosen by the woman’s age and tumor test findings, including Chemotherapy, Hormone therapy and HER2 targeted drugs [14].
Stage IIB	Tumor is larger, ranging in size from a walnut to a lime.	
Stage III	Although cancer has not progressed to the bones or organs, it is considered advanced and more difficult to treat [15].	
Stage IIIA	Indicates that cancer discovered in up to nine lymph nodes that run from your armpit to your collarbone in a chain. It may have spread to or swollen the nodules deep within your breast. There is a huge tumor in the breast in some cases, yet there is no tumor in others even if the tumor hasn’t migrated to the lymph nodes.	There are two main approaches to treating stage III breast cancer:Neoadjuvant chemotherapy (before surgery). Trastuzumab (Herceptin), a targeted medication for HER2-positive malignancies, is also used, sometimes with pertuzumab (Perjeta). This may cause the tumor to shrink to the point where a woman may receive breast-conserving surgery (BCS) [15]. A mastectomy is going to perform if the tumor does not shrink enough. Lymph nodes in the area will also need to be examined. For stage III malignancies, a sentinel lymph node biopsy (SLNB) is typically not an option. Instead, an ALND is usually performed. After surgery, radiation treatment is often required. Additional chemo may be administered following surgery in rare circumstances. Some women with HER2-positive malignancies will be treated for up to a year with trastuzumab (with or without pertuzumab) after surgery. Many women with HER2-positive malignancies will be treated with trastuzumab (with or without pertuzumab) for up to a year, followed by surgery and additional trastuzumab (with or without pertuzumab). If any residual cancer is identified at the time of surgery following neoadjuvant treatment, trastuzumab may be switched to a new medicine called ado-trastuzumab emtansine, which is given every three weeks for 14 doses [16]. If hormone receptor-positive cancer in lymph nodes and have finished a year of trastuzumab, in such condition doctor may propose therapy with neratinib, an oral medication. Adjuvant hormone treatment will be given to women with hormone receptor-positive (ER-positive or PR-positive) breast tumors, which may usually be administered simultaneously as trastuzumab.
Stage IIIB	Indicates that it has spread into the chest wall or skin around your breast.	
Stage IV	Breast cancer cells have gone beyond the breast and into the lymph nodes surrounding it. Skeletal bones, lungs, liver, and brain are the most prevalent locations. The term “metastatic” refers to the fact that cancer has moved beyond the part of the body where it has been initially discovered [16].	Chemotherapy is a treatment for advanced-stage breast cancer that kills or damages as many cancer cells as possible. Chemotherapy is often used to treat advanced-stage breast cancer since it affects the whole body. Newer chemotherapy medications, such as Taxol (paclitaxel), Abraxane (albumin-bound or nab-paclitaxel), Taxotere (docetaxel), Adriamycin (doxorubicin), Ellence (epirubicin), and Halaven (erubilin), have been found to help women with advanced-stage breast cancer survive longer. Gemzar (chemical name: gemcitabine), Xeloda (chemical name: capecitabine), Navelbine (chemical name: vinorelbine), and Ixempra are some of the other chemotherapies utilized in metastatic breast cancer (chemical name: ixabepilone) [16,17].

**Table 2:** Molecular subtypes of breast cancer and their current conventional drug therapy.

Molecular Sub Type	Indication	Other Condition	HarmoneTherapy	Chemotherapy	Anti-HER2 (Trastuzu-Mab)
Luminal A	The most prevalent molecular type, Luminal A tumors, develops at a slower pace than other cancers [18].	Low tumor burden	Yes	No	No
		High tumor burden * or grade 3	Yes	Yes	No
Luminal B	More aggressive than luminal A cancer cells because they develop quicker.	HER2+	Yes	Yes	Yes
		HER2−	Yes	Yes	No
Triple-negative breast cancer (TNBC)	TNBC was more likely to occur in females with early menarche, larger waist-to-hip ratio, greater fecundity, shorter duration of breastfeeding, higher body mass index, and became more prevalent in premenopausal patients, according to several epidemiological studies.	Not applicable	No	Yes	No
HER2-positive	Cancers tend to grow faster than luminal cancers and can have a worse prognosis, but they are often success-fully treated with targeted therapies aimed at the HER2 protein.	Not applicable	No	Yes	Yes
Normal-like breast cancer	It closely matches luminal A (Breast cancer is a diverse disease at the molecular level, with activation of the HER2, stimulation of hormone receptors (estrogen receptor and progesterone receptor), and/or BRCA mutations being the most common molecular hallmarks.) Treatment options vary depending on the molecular subtype. Conventional cancer chemotherapies have limitations, such as drug resistance and non-differentiation between healthy and malignant cells, which leads to significant side effects and systemic toxicity [18].				

Note: * ≥4 positive LN, T3 or higher; Abbreviations: HER2, human epidermal receptor 2; LN, lymph nodes.

**Table 3:** Breast cancer drugs approved by the US Food and Drug Administration.

Generic Name or Brand Name	Background	Molecular Structure	Stage	Dosage &Strength	Route	References
Trastuzumab (Herceptin)	Trastuzumab is a recombinant IgG1 kappa, humanized monoclonal antibody that selectively binds with high affinity in a cell-based assay (Kd = 5 nM) to the extracellular domain of the human epidermal growth factor receptor protein (HER2).	C_6470_H_10012_N_1726_O_2013_S_42_	HER2+	Powder, for solution (440 mg/ vial)	Iv	[13,14]
				Injection, powder, for solution (150 mg, 150 mg/7.4 mL).		
				Injection, solution (600mg)	Sc	
Pertuzumab (Perjeta)	Pertuzumab is an antineoplastic agent used in the treatment of HER2-positive metastatic breast cancer in combination with other antineoplastic agents.	C_17_H_27_NO_2_	Metastatic HER2+ in patients who have not been treated with hormone therapy or chemotherapy.	Injection, solution, concentrate (420 mg)	Iv	[15]
			As neoadjuvant therapy in patients with locally advanced, inflammatory, or early-stage cancer; as adjuvant therapy in patients with early-stage cancer who have a high recurrence risk	Pertuzumab (420 mg/14 mL) + Trastuzumab (440 mg/vial),Powder, for solution; Solution.	Iv	
				Pertuzumab (1200 mg) + Trastuzumab (600 mg), Injection, Solution.	Sc	
				Pertuzumab (1200 mg/15 mL) + Hyaluronidase (human recombinant) (30,000 U/15 mL) + Trastuzumab (600 mg/15 mL), Injection, Solution.	Sc	
Ado-Trastuzumab Emtansine (Kadcyla)	Ado-Trastuzumab emtansine, is a first-in-class HER2 antibody drug conjugate (ADC) composed of Genentech’s trastuzumab antibody and ImmunoGen’s cell-killing chemical, DM1.	C_6448_H_9948_N_1720_O_2012_S_44_•(C_47_H_62_C_l_N_4_O_13_S)n	Used in patients with HER2-positive metastatic breast cancer who have previously received taxane and/or trastuzumab for metastatic cancer or who had a recurrence of their cancer within six months after adjuvant therapy.	Injection, powder, lyophilized, for solution (20 mg/1 mL, 100 mg)	Iv	[16,17,18]
				Powder, for solution (160 mg/vial).		
Abemaciclib (Verzenio)	Abemaciclib is an antitumor agent and dual inhibitor of cyclin-dependent kinases 4 (CDK4) and 6 (CDK6) that are involved in the cell cycle and promotion of cancer cell growth in case of unregulated activity.	C_27_H_32_F_2_N_8_	As Monotherapy: Advanced or metastatic HR+ and HER2−	Starting dose: - Combination therapy: 150 mg 2 times a day - Monotherapy: 200 mg orally 2 times a day - First dose reduction: - Combination therapy: 100 mg tablet 2 times a day - Monotherapy: 150 mg tablet orally 2 times a day - Second dose reduction: - Combination therapy: 50 mg tablet 2 times a day - Monotherapy: 100 mg tablet 2 times a day - Third dose reduction: - Combination Therapy: N/A - Monotherapy: 50 mg tablet 2 times a day	Oral	[18,19]
			In combination with an aromatase inhibitor: Initial endocrine-based therapy for postmenopausal women with hormone receptor (HR)-positive, human epidermal growth factor receptor 2 (HER2)-negative advanced or metastatic breast cancer			
			In combination with fulvestrant: For women with hormone receptor (HR)-positive, (HER2)-negative advanced or metastatic breast cancer with disease progression following endocrine therapy			
Cyclophosphamide (Cytoxan, Endoxan, Cycloblastin, Neosar, Revimmune)	Precursor of the alkylating nitrogen mustard anticancer and immunosuppressive drug aldophosphamide, which must be activated in the liver to create the active aldophosphamide	C_7_H_15_Cl_2_N_2_O_2_P	Advanced or metastatic	Injection, powder, for solution (2 g/100 mL)	Iv; Oral	[19,20]
				Tablet (25 mg/L)	Oral	
				Capsule (25 mg/L)	Oral	
Docetaxel (Taxotere)	Docetaxel is a well-known anti-mitotic chemotherapy drug that is mostly used to treat breast, ovarian, and non-small cell lung cancer. Docetaxel binds to tubulin reversibly and with a high affinity in a 1:1 stoichiometric ratio.	C_43_H_53_NO_14_	Metastatic cancer that has not gotten better with other chemotherapy or node positive cancer removed by surgery	Injection (10 mg/1 mL, 80 mg/4 mL, 160 mg/8 mL)	Iv	[19,21]
				Injection, solution, concentrate(20 mg/1 mL)		
Exemestane (Aromasin)	Exemestane is an oral steroidal aromatase inhibitor used in the adjuvant treatment of hormonally-responsive breast cancer in postmenopausal women. It irreversibly binds to the active site of the enzyme resulting in permanent inhibition.	C_20_H_24_O_2_	Early stage, advanced or ER+.	Tablet (25 mg)	Oral	[19,22]
Tamoxifen Citrate	Tamoxifen is a selective estrogen receptor modulator used to treat estrogen receptor positive breast cancer, reduce the risk of invasive breast cancer following surgery, or reduce the risk of breast cancer in high risk women.	C_26_H_29_NO	Advanced or metastatic ER+	Nolvadex:Tablet(20 mg/L, 10 mg/L)	Oral	[19,23]
				Tamofen:Tablet (10 mg, 20 mg)		
				Soltamox:Liquid(20 mg/10 mL or 10 mg/5 mL)		
				Tamone:Tablet (10mg)		
Anastrazole (Arimidex)	Anastrozole is a non-steroidal aromatase inhibitor (AI) comparable to letrozole that is used to treat postmenopausal women with estrogen-responsive breast cancer.	C_17_H_19_N_5_	Early-stage, HR+ in women who have already received other treatment; HR+ locally advanced or metastatic breast cancer or hormone receptor unknown; advanced breast cancer that has gotten worse after treatment with tamoxifen citrate	Tablet, film coated (1 mg/mL)	Oral	[19,24]
				Pellet Anastrozole (20 mg/L) + Testosterone (200 mg/L)	Oral	
				Pellet, implantable Anastrozole (4 mg/L) + Testosterone (60 mg/L)	Sc	
Doxorubicin	Doxorubicin is a cytotoxic anthracycline antibiotic isolated from cultures of Streptomyces peucetius var. caesius.	C_27_H_29_NO_11_	Node-positive cancer removed by surgery	Adriamycin: Solution (2 mg/mL)	Iv; Ives	[19,25]
				Caelyx: Injection, solution, concentrate (2 mg/mL)	Iv	
Methotrexate (Rheumatrex, Trexall)	Methotrexate is a folate derivative that inhibits several enzymes responsible for nucleotide synthesis. This inhibition leads to suppression of inflammation as well as prevention of cell division. Because of these effects, methotrexate is often used to treat inflammation caused by arthritis or to control cell division in neoplastic diseases such as breast cancer and non-Hodgkin’s lymphoma	C_20_H_22_N_8_O_5_	Advanced or metastatic	Tablet (2.5 mg/L)	Oral	[19,26]
				Solution (25 mg/mL)	Im; Ia; Iv	
Vinblastine Sulfate (Velban)	Antitumor alkaloid isolated from Vinca rosea and used to treat breast cancer, testicular cancer, neuroblastoma, Hodgkin’s and non-Hodgkins lymphoma, mycosis fungoides, histiocytosis, and Kaposi’s sarcoma	C_46_H_58_N_4_O_9_	Advanced or metastatic	Solution (1 mg/1 mL)	Iv	[19,27]
Thiotepa (Thioplex)	Alkalyting agent and is mostly used to treat breast cancer, ovarian cancer, and bladder cancer. It is also used as conditioning for bone marrow transplantation. Its main toxicity is myelosuppression.	C_6_H_12_N_3_PS	Advanced or metastatic	Injection, powder, for solution (100 mg; 15 mg; 30 mg)	Ic; Iv; Ives	[28]
Fulvestrant (Faslodex)	Fulvestrant is an estrogen receptor antagonist used to treat HR+ breast cancer that may also be HER2−.	C_32_H_47_F_5_O_3_S	HR+ and HER2− advanced cancer that has not been treated with hormone therapy; HR+ advanced cancer that got worse after treatment with hormone therapy or combined; used with palbociclib or abemaciclib in women with HR+ and HER2− advanced or metastatic cancer that got worse after treatment with hormone therapy	Injection, solution (50 mg/1 mL or 250 mg/5 mL)	Im	[29]
Paclitaxel (Abraxane)	Paclitaxel is a taxoid chemotherapeutic agent isolated from the bark of the Pacific yew tree, used as first-line and subsequent therapy for the treatment of advanced carcinoma of the ovary, and other various cancers including breast and lung cancer.	C_47_H_51_NO_14_	Recurrent or metastatic	Injection, solution (6 mg/1 mL, 30 mg/5 mL, 300 mg/50 mL)	Iv	[16,17,30]
Gemcitabine Hydrochloride(Gemzar)	Gemcitabine is a nucleoside metabolic inhibitor used as adjunct therapy in the treatment of certain types of ovarian cancer, non-small cell lung carcinoma, metastatic breast cancer, and as a single agent for pancreatic cancer.	C_9_H_11_F_2_N_3_O_4_	Combined with paclitaxel in cancer that has not gotten better with other chemotherapy	Injection, solution (38 mg/1 mL, 200 mg/vial, 1 g/vial, 2 g/vial)	Iv	[31]
Letrozole (Femara)	Letrozole is an aromatase inhibitor used to treat breast cancer in postmenopausal women.	C_17_H_11_N_5_	Early-stage HR+ in women who have already received other treatment; early-stage cancer that has been treated with tamoxifen citrate for at least five years; locally, advanced or metastatic HER2+ and HR+ or HR−; advanced cancer that has gotten worse after anti-estrogen therapy.	Tablet, film coating (2.5 mg)	Oral	[32]
				Letrozole (2.5 mg/L) + Ribociclib succinate (200 mg/L)		
Olaparib (Lynparza)	Olaparib is a chemotherapeutic agent used to treat recurrent or advanced ovarian cancer and metastatic breast cancer in patients with specific mutations and prior history of chemotherapy.	C_24_H_23_FN_4_O_3_	Metastatic HER2− with certain mutations in the BRCA1 or BRCA2 genes in patients who have been treated with chemotherapy given before or after surgery.	Tablet, film coated (100 mg, 100 mg/L, 150 mg, 150 mg/L)	Oral	[33]
				Capsule (50 mg, 50 mg/L)		
Epirubicin (Ellence)	Epirubicin is an anthracycline topoisomerase II inhibitor used as an adjuvant to treating axillary node metastases in patients who have undergone surgical resection of primary breast cancer.	C_27_H_29_NO_11_	Node-positive breast cancer removed by surgery	Injection, solution (2 mg/1 mL)	Iv	[34]
Eribulin Mesylate (Halaven)	Eribulin is a microtubule inhibitor used to treat metastatic breast cancer and metastatic or unresectable liposarcoma.	C_40_H_59_NO_11_	Patients who have been treated with anthracycline and taxane	Injection, solution (0.44 mg/mL, 0.5 mg/1 mL)	Iv	[35]
Capecitabine (Xeloda)	Capecitabine is an orally-administered chemotherapeutic agent used in the treatment of metastatic breast and colorectal cancers. Capecitabine is a prodrug, that is enzymatically converted to fluorouracil (antimetabolite) in the tumor, where it inhibits DNA synthesis and slows growth of tumor tissue.	C_15_H_22_FN_3_O_6_	Metastatic cancer that has not gotten better with other chemotherapy	Tablet, film coated (150 mg, 500 mg)	Oral	[36]
Ixabepilone (Ixempra)	Ixabepilone is a microtubule inhibitor administered in combination with capecitabine or alone in the treatment of metastatic or locally advanced breast cancer that has shown inadequate response to taxanes and anthracyclines.	C_27_H_42_N_2_O_5_S	Locally advanced or metastatic cancer that has not gotten better with other chemotherapy	Kit (15 mg/15 mg, 45 mg/45 mg)	Iv	[37]
Palbociclib (Ibrance)	Palbociclib is an endocrine-based chemotherapeutic agent used in combination with other antineoplastic agents to treat HER2-negative and HR-positive advanced or metastatic breast cancer.	C_24_H_29_N_7_O_2_	Recurrent or metastatic	Tablet, film coated (75 mg, 100 mg, 125 mg/L)	Oral	[38]
				Capsule (75 mg, 75 mg/L, 125 mg/L)		
Ribociclib (Kisqali)	Ribociclib is a kinase inhibitor used to treat HR+, HER2− advanced or metastatic breast cancer.	C_23_H_30_N_8_O	Recurrent or metastatic	Tablet, film coated (200 mg, 200 mg/L)	Oral	[39]

**Table 4:** Various challenges associated with breast cancer drug therapy and the ways nanomedicine can be used to tackle these challenges.

Challenges to Breast Cancer Drug Therapy	How Nanomedicine Can Help
1.Low specificity for breast cancer	Nanomedicine uses passive and active targeting to enhance tumor medication levels while decreasing drug levels in noncancerous cells.
2. Undesirable pharmacokinetics such as quick clearance and short half-life	Use of strategies such as PEGlyation to extend the circulation time.
3. Anticancer drugs or excipients, such as surfactants and organic co-solvents, have dose-limiting toxicity.	Tumor progression selectivity; regulated medication release from nanocarrier; solvent- and surfactant-free nanoformulation.
4. Drug resistance at cellular level, for example, increased drug efflux transport	Both passive and active targeting may improve endocytosis; some nanoformulations may block drug efflux processes; and co-delivery of medicines that target drug resistance mechanisms may improve endocytosis.
5. Lower pH, hypoxia, cancer microenvironment interaction, and other factors contribute to drug resistance in the tumour microenvironment.	Targeting tumor microenvironment; use of stimulus-responsive nanoformulations such as pH-responsive devices.
6. Difficulty in eradicating cancer stem cells	Targeting cancer stem cells.

Abbreviation: PEG, polyethylene glycol.

**Table 5:** Advantages and disadvantages of different nanomaterials for breast cancer treatment.

Nanocarrier	Advantages	Disadvantages	Reference
Liposome	Uses for a wide variety of drugs and capable of increasing drug load while reducing unwanted drug activity.	Toxicity is caused by cationic lipids. The mononuclear phagocyte system degrades the nanocarriers quickly.	[56]
Dendrimer	It has higher loading capacity due to a variety of multifunctional surface groups and intracellular cavities, as well as it has high bioavailability.	Rapid clearance, organ accumulation, synthesis variability	[56]
Micelles	Reduction of toxicity and other adverse effects.	Use only for lipophilic drugs, low drug loading capacity	[56,57]
Carbon nanotube	To deliver chemotherapeutic and imaging agents, it must be capable of penetrating and localize at the cellular level.	Potential material toxicity	[57]
Polymeric nanoparticles	These are biocompatible, biodegradable, nontoxic, have a longer blood circulation time, less drug change, are less reactive to enzymatic degradation, and site-targeted administration.	Degradation of the carrier	[58]
Solid lipid nanoparticles (SLNs)	Due to its organic nature, it has a high solubility and bioavailability.		
	The kinetics of medication release can be better controlled.	Low drug loading capacities Possibly containing other colloidal structures and complex physical state	[59]
Nanostructured lipid carrier (NLCs)	It is second generation SLNs having high drug loading and entrapment potential.		
	Long-term stability, prevent particles from coalescing, low toxicity, biodegradation, drug protection.	Gelation of lipid dispersion Polymorphic transition	[59]
	Biocompatible to a high degree.		
	Organic solvents may be avoided since the procedures are water-based.		
	Simple to scale-up and sterilize, and they are less costly than other materials.		
	Carriers based on polymers or surfactants.		
	Improve drug release control and/or target.		
	When compared to other NLCs, NLCs provide excellent and greater medication content.		
	NLCs may transport both lipophilic and hydrophilic molecules. Biodegradability of the majority of lipids.		

**Table 6:** Cancer nanomedicine liposomal formulation in the treatment or management of breast cancer.

Brand Name	Therapeutic Agent	Clinical Trial Phase	Company Name	Reference
Doxil	DOX	Approved	Sequus Pharmaceuticals Inc.	[68]
LipoDox	DOX	Approved	Sun Pharmaceutical Industries Ltd.	[69]
dHER2+AS15	HER2 antigen	Phase 1/Phase 2	GlaxoSmithKline	[75]
Lipoplatin	Cisplatin	Phase III	Regulon Inc.	[71]
EndoTAG1	Paclitaxel	Completed	MediGene	[72]
DPX-0907	Multicancer-associated antigens	Completed	ImmunoVaccine Technologies	[73,74]
LEM-ETU	Mitoxantrone	Phase I	NeoPharm Inc	[75,76]
Myocet™	DOX	Approved	Elan Pharma	[77]
LEP-ETU	Paclitaxel	Phase II	NeoPharm Inc	[78]
ThermoDox™	DOX	Phase III	Celsion	[79]
MM-302	DOX	Phase 1	Merrimack Pharmaceuticals	[80]

**Table 7:** Cancer nanomedicine dendrimeric formulation in the treatment or management of breast cancer.

Brand Name	Therapeutic Agent	Clinical Trial Phase	Company Name	Reference
DEP^®^ docetaxel	Docetaxel	Phase I	Starpharma	[90]
MAG-Tn3	Vaccine composed of tri Tnglycotope	Phase I	Institute Pasteur	[85]

**Table 8:** Cancer nanomedicine micelle formulation in the treatment or management of breast cancer.

Brand Name	Therapeutic Agent	Clinical Trial Phase	Company Name	Reference
Genexol-PM™	Paclitaxel	Phase II	Samyang	[100]
NC-6300	Epirubicin	Phase I	Nanocarrier Co.	[101]
NK911	Doxorubicin	Phase II	Nippon Kayaku Co.	[102]
NK105	Paclitaxel	Phase III	Nippon Kayaku Co.	[103]

**Table 9:** Cancer nanomedicine Polymeric Nanoparticles formulation in the treatment or management of breast cancer.

Brand Name	Therapeutic Agent	Clinical Trial Phase	Company Name	Reference
NCT00629499 P	Paclitaxel/Cyclophosphamide	Phase II	SCRI DevelopmentInnovations, LLC	[119]
NCT04249167	Cryoablation, atezolizumab/nab-paclitaxel	Early Phase I	Mayo Clinic	[120]
NCT03606967	Paclitaxel and durvalumab with or without neoantigen vaccine	Phase II	National Cancer Institute (NCI)	[121]
NCT00407888	Doxorubicin hydrochloride, cyclophosphamide, and filgrastim followed by paclitaxel	Phase II	University of Washington	[122]
NCT00616967	Carboplatin and nab-paclitaxel with or without vorinostat	Phase II	Sidney Kimmel Comprehensive Cancer Center at Johns Hopkins	[123]

**Table 10:** List of excipients commonly used in the manufacture of SLNs.

Lipid	Surfactant	HLB Value
Mixtures of mono-, di- and triglycerides Witeposol bases Glyceryl monostearate (Imwitor900), Glyceryl behenate (Compritol888ATO) and Glycerylpalmitosterate (PrecirolATO5)	Polysorbate80	15
	Sodium cholate	18
	Sodium glycocholate	14.9
	Cetylpyridiniumchloride	15
	Sodium dodecylsulphate	40
	Sodiumoleate	18
	Polyvinylalcohol	15–19
	CremophorEL	12–14
Waxes Beeswax and Cetylpalmitate	Lecithin	4–9
	Poloxamer188	29
Solid fatty acids Stearic acid, Palmitic acid and Behenic acid	Poloxamer407	21.5
	Tyloxapol	-
Other lipids Miglyol812 and Paraffin	Polysorbate20	16.7
	Polysorbate60	14.9

**Table 11:** Various SLNs formulations and their intervention in the treatment or management of breast cancer.

Cytotoxic Agents	Method of Preparation	Interventions	Reference
Silymarin	Hot homogenization method	Reduced A549 and MCF-7 cell proliferation induced apoptosis in both cells and increased bioavailability. In mammary carcinogenesis	[132]
Chitosan encapsulating Docetaxel	Hot homogenization method	Particle size was increased from 143 ± 2.5–225 nm ± 3.6, and the surface charge was reversed from 35 ± 3.3 to 25 mV± 2.1. Slower drug release, increased cytotoxicity in vitro, and tumor inhibition	[133]
Letrozole (Folic acid targeted)	Solvent emulsification evaporation	Enhanced biocompatibility and triggering apoptosis in a threat manner with low systemic adverse effects. In hormone-dependent breast cancer	[134]
Tamoxifen citrate (Transferrin targeted)	Hot emulsification method	Tamoxifen citrate increased targeting affinity towards breast cancer cells MCF-7 substantiated the developed SLN’s potential for breast cancer treatment. In ER+ breast cancers	[135]
Docetaxel	High-energy method	PS and PDI were 128 nm and 0.2 with a negative zeta potential with 86% encapsulation, 2% drug loading, and a regulated drug-release profile. In vivo investigations revealed that SLN-DTX had a greater anticancer activity by decreasing tumor volume. In Metastatic breast cancer	[136]
Docetaxel palmitate (DTX-PL)	Micro-emulsification technique	Oral bioavailability is improved, with a long biological half-life. The increased cytotoxicity in MDR cancer cells supports the promise of the novel lipophilic compound, which has improved the drug’s overall performance. In TNBC	[137]
Gefitinib	Modified hot homogenization method	SLNs were nanosized (90 percent) within 72 h, according to SEM images. The Higuchi model matches the kinetic analyses of GFT-SLNs (R2 = 0.935). The Higuchi model matches the kinetic analyses of GFT-SLNs (R2 = 0.935). In vitro assays of SLN showed significantly stronger antitumor activity (cell survival >65%) than free drug (*p* < 0.05). It has a high antitumor activity as well as improved drug dispersion in tissues.	[138]
Annona muricata fruit extract	High-pressure homogenization followed by ultrasonication method.	PS and percent EE were reported to be 134.8 nm and 83.26%, with a CDR of 79.83% after 48 h. It had an apoptotic impact and was more effective in killing MCF7 cancer cells.	[139]
Doxorubicin An arginine-glycine-aspartic (RGD) tripeptide modified, pH-sensitive (RGD-DOX-SLNs)	Emulsification and low-temperature solidification method	AUC –time curve was 5.58 times higher. T1/2 and Cmax were 10.85 h and 39.12 L/kg/h. *in vitro* and in vivo revealed that RGD-DOX-SLNs could be a potential new lipid carrier that might enhance breast cancer therapy. In Metastatic breast cancer	[140]
Curcumin	Emulsification evaporation-low temperature solidification method	Drug loading and encapsulation efficiency in SLNs were 23.38% and 72.47%. Greater cytotoxicity against SKBR3 cells. In an in vitro cellular uptake study, it found to have good absorption efficiency by SKBR3 cells. Cur-SLNs also produced higher apoptosis in SKBR3 cells. Decreased the manifestation of cyclin D1 and CDK4. These data suggest that Cur-SLNs might be a promising chemotherapeutic formulation for breast cancer therapy.	[141]
Pomegranate extract	Hot homogenization followed by the ultra-sonication technique	Improves bioefficacy, especially in MCF-7 breast cancer cells, where the IC50 was lowered by 47-fold from 49.2 to 1.05 g/mL and it has cytotoxicity in cancer cells vs normal cells Pomegranate extract has promising agent, especially for breast cancer. In Metastatic breast cancer	[142]
Talazoparib	Hot homogenization method	Talazoparib SLNs are more effective than talazoparib at suppressing MDR1, BCRP, and MRP1 genes and protein expression levels. Reverse MDR-mediated resistance in TNBC.	[143]
Resveratrol	Emulsification and low-temperature solidification method.	Res-SLNs were shown to be more effective at stopping MDA-MB-231 cells from proliferating andhad a considerably higher inhibitory impact on MDA-MB-231 cell invasion and migration. Res-SLNs increased the ratio Bax/Bcl-2 but lowered the expression of cyclinD1 and c-Myc, according to Western blot examination. Res–SLN has a lot of potential as a breast cancer therapy.	[131]

**Table 12:** List of excipients commonly used in the manufacture of NLCs.

Lipid			Surfactant and Co-Surfactant
Solid Lipid		Liquid Lipid	
Mixtues of mono, di andtriglycerides	Monoglycerides: Caprylate triglyceride, Caprate triglyceride, Glyceryl and tribehenate/Tribehenin. Diglycerides: Glyceryl palmitostearate and Glyceryl dibehenate. Triglycerides: Caprylate triglyceride, Caprate triglyceride, Glyceryl and tribehenate/Tribehenin.	Soya bean oil Oleic acid Medium chain triglycerides (MCT) caprylic- and capric triglycerides, αtocopherol Vitamin E Squalene Hydroxyoctacosanylhydroxystearate, Transcutol and Isopropyl myristate.	Poloxamer 188 Poloxamer 407 Soyabean phosphatidylcholin, Lecithin Tween 80 Cremophor^®^ RH40, Sodium taurodeoxycholate, Sodium oleate Sodium dodecyl sulphure Butanol, Butyric acid
Waxes	Cetyl Palmitate, Carnauba, and wax Beeswax.		
Fatty acids	Dodecanoic acid, Myristic acid, Palmitic acid and Stearic acid		

**Table 13:** Various NLC formulations prepared by different methods and their intervention in the treatment or management of breast cancer.

Nanocarrier	Method of preparation	Interventions	Reference
Cabazitaxel (*CBZ-loaded NLCs)*	Hot homogenization method	NLCs containing CBZ induced a 6- and 2.5-fold increase in cytotoxicity, as well as an increase in apoptosis. In vitro cell culture assays, MDA-MB-468 and MCF-7 cells had reduced motility. In cells, NLC absorption was 2.5 to 2.1 times that of CBZ alone. For tumors that are resistant to drugs.	[151]
Luteolin (LTN)-encapsulated chitosan (CS) (LTN-CS-NLCs)	Melt emulsification ultrasonication technique	Mucoadhesion, gastro-intestinal stability, and intestinal penetration were all significantly improved in LTN-CS-NLCs. MDA-MB-231 and MCF-7 cells showed improved antioxidant activity as well as dose and time-dependent cytotoxicity. LTN-NLCs coated with chitosan show a lot of potential in the treatment of Breast cancer.	[152]
NLC loaded with Imatinib (NANIMA)	Hot homogenization method	The particle size of 104.63 ± 9.55 d.nm, PDI of 0.227 ± 0.06, and EE of 99.79 ± 0.03 and was sustained released. In cytotoxicity experiments on MCF-7 breast cancer cells, optimum NANIMA (IC50 = 6 M) was shown to be 8.75 times more effective than IMA alone (IC50 = 52.5 M). For the treatment of breast cancer, a lower dose of IMA-rich NLC will suffice rather than IMA alone. Furthermore, NANIMA has less adverse effects than IMA alone, leading in a satisfactory therapeutic outcome in the treatment of breast cancer.	[153]
Raloxifene	Ultrasonication method	*In vitro*, the RLN-NLCs were more cytotoxic to MCF-7 cells than the RLN solution. An *ex vivo* intestinal systemic absorption analysis revealed that the RLN-NLCs had better intestinal permeability. When RLN-NLCs were compared to RLN solution in an in vivo pharmacokinetic investigation in female Wistar rats, the oral bioavailability of RLN from RLN-NLCs increased 4.79-fold. One of the paths for a novel nanotherapeutic approach to the treatment of Breast cancer.	[154]
Curcumin-Loaded Magnetic Lipid Nanoparticles (CUR-NLC-SPIONs)	SPIONs by co-precipitation followed by CUR-NLC-SPIONs by homogenization technique	The average PS was 166.7 ± 14.20 nm, with a mean ZP- −27.6 ± 3.83 mv, PDI of 0.24 ± 0.14, EE was 99.95 ± 0.015%, and drug-loading capacity was 3.76 ± 0.005%. CUR-NLC-SPIONs had a more substantial cytotoxic effect against human breast cancer cells than free CUR. This new drug delivery technology, which uses superparamagnetic properties, might be utilized to create new biocompatible drug carriers and tailored cancer therapies.	[155]
Curcumin	High shear hot homogenization method	The small mean PS, spherical shape and negative ZP of NLCs assisted their internalization into cells. By regulating and suppressing P-gp expression, glyceryl monooleate enhanced the cytotoxic effects of CUR.	[156]
Docetaxel-loaded NLCs functionalized with trastuzumab (Herceptin)	Solvent extraction technique followed by probe sonication.	DTX added in chemically connected NLCs to Herceptin had more cytotoxic effects than physically coated nanoparticles. The Herceptin conjugated NLCs seem to have the potential for delivering DTX to HER2-positive breast cancer cells in a targeted way.	[157]

**Table 14:** Combinatorial approach of natural agent with synthetic drug in various nanocarriers for breast cancer treatment.

Combinatorial Approach	Nanocarrier	Interventions	Reference
DOX & Acridine orange (AO)	Gold core silica shell (AuMSS) nanosphere	AuMSS nanospheres have particle size of 192.6 ± 2.9 nm. AuMSS nanoparticles functionalization with the PANIS silane derivatives (TPANIS) promoted a slight increase in the nanoparticles size and colloidal stability. Additionally, both the DOX and AO were successfully encapsulated on the AuMSS-TPANIS nanospheres. Moreover, the AuMSS nanospheres functionalization with TPANIS significantly increased their internalization by MCF-7 cells resulting in an enhanced cytotoxic effect.	[165]
DOX and Crocin (carotenoid)	PLGA nanoparticles (PDCR NPs)	The PDCR NPs had a particle size of 174.2 ± 1.57 nm and showed a sustained and controlled release pattern. PDCR NPs cause apoptosis in breast cancer cells by reducing reactive oxygen species (ROS) and altering mitochondrial potential, resulting in cell-cycle arrest in the G2/M phase and death. In tumor-induced animal investigations, PDCR NPs showed decreased tumor volume when compared to control groups. In vitro and in vivo, the co-delivery of natural anticancer bioactive crocin with doxorubicin in PDCR NPs presents a feasible controlled-release nanoplatform for effective drug administration.	[166]
Tamoxifen (TAM) and sulforaphane (SFN)	TAM-SFN NLCs	TAM-SFN-NLCs exhibited a particle size of 121.9 ± 6.42 nm and zeta potential of −21.2 ± 2.91 mV. Oral bioavailability is improved. In vivo study revealed that SFN significantly reduced TAM-related toxicity.	[167]
Stearic acid loaded with capsaicin	SLNs	Synthesized SLNs were predominantly spherical, 80 nm diameter particles that proved to be biocompatible with good stability in aqueous conditions.In vivo biodistribution showed that 48h. The IC_50_ of capsaicin-loaded SLNs in HepG2 cells in vitrowas 21.36 μg× mL^−1^ and enhanced anticancer activity.	[168]
Variabilin Loaded Stearic Acid (Var-SLNs)	SLNs	Var-SLNs triggered apoptosis in HT-29, MCF-7, and PC-3 cells at rates of 47 percent (vs. 38% for variabilin), 48 percent (vs. 29% for variabilin), and 59% (vs. 29% for variabilin). An average size of 83.5 nm. Both variabilin and Var-SLN revealed comparable activity to Ceramide against the MCF-7 breast cancer cell line, revealing IC_50_ values of 34.8, 38.1 and 33.6 μM for variabilin, Var-SLN and Ceramide, respectively. Encapsulation into SLNs also has a “protective effect” on the non-tumorigenic epithelial cell line (MCF12A). Incorporating unstable or poorly soluble medicines into SLNs might save molecules that were previously thought to be druggable owing to poor physicochemical properties.	[169]
Epigallocatechin-gallate (EGCG) with Bombesin	SLNs	The bombesin-conjugated nanoparticles (EB-SLN) had a diameter of 163.4 ± 3.2 nm. IC_50_ values for EGCG, EGCG-SLN and EB-SLN in MDA-MB-231 were 65.4 ± 4.9 μg/mL, 6.9 ± 1.1 μg/mL and 3.2 ± 1.7 μg/mL, Similarly, these formulations were also more effective against B16F10 cells with IC50 values for EGCG, EGCG-SLN and EB-SLN found to be 59.3 ± 6.4 μg/mL,28.2 ± 1.9 μg/mL and 15.6 ± 1.3 μg/mL, respectively. It revealed that peptide-conjugated formulations had higher cytotoxicity against cancer cell lines than non-conjugated formulations. Furthermore, in-vivo investigations on C57/BL6 mice revealed that animals treated with the compound had a higher survival rate and had a smaller tumor volume. These findings support the system’s promise as a unique and effective drug delivery mechanism in breast cancer treatment.	[170]
DOX + CUR	Micelle	DOX + CUR-Micelles showed a consistent particle size, strong encapsulation effectiveness, a long-term release profile, and good colloidal stability. DOX + CUR-Micelles had the greatest cytotoxicity and cell apoptosis-inducing properties against DOX-resistant MCF-7/Adr cells in an in vitro cytotoxicity investigation. Furthermore, DOX + CUR-Micelles increased the cellular absorption of DOX through energy-dependent and caveolae-mediated endocytosis, and dramatically reversed MDR effects via CD44 targeted delivery and the synergic action of released CUR. In vivo data showed that DOX + CUR-Micelles not only had superior tumour accumulation and tumour targeting, and more effectively reduced tumour development in 4T1 tumor-bearing mice. In conclusion, this targeted combinational micellar delivery system including DOX and CUR might be a viable tumour therapeutic vehicle.	[171]
CUR and paclitaxel	PEGylated lipid bilayer coated mesoporous silica nanoparticles (PLMSNs)	MSNs with pore diameter of 2.754 nm and particle size of 115 ± 15 nm. In vitro release tests revealed that PLMSNs enhanced PTX dissolution when compared to PTX powder suspension and had a longer release time. PTX-CUR-PLMSNs showed a definite and long-lasting cytotoxic impact on canine breast cancer cells. This increased and extended activity of PTX-CUR-PLMSNs may contribute to the impact of sustained release.	[172]
	Micelle	The obtained IC_50_ values of the mPEG-PBLA-PVIm triblock copolymer (PPBV). Micelles/PTX + CUR at pH 6.5 were 0.47 mg/mL (PTX) and 1.22 mg/mL (CUR), respectively. Tumor growth inhibition with no substantial recurrence. Furthermore, systemic administration of PPBV micelles/PTX+ CUR exhibited superior tumor inhibition activity and bCSCs-killing capacity in vivo. Consequently, collaborative therapy of PTX and CUR using pH multistage responsive PPBV micelles could be a potential strategy for inhibiting breast tumor growth by simultaneously eliminating bCSCs and non-CSCs.	[173]
	Nanoliposomes (CL-APNs)	The particle size of CL-APNs was found to be 252.13 ± 5.055nm. In vitro, drug-loaded nanoliposomes have a sustained drug release profile and relative ratio of migration was 37.64 ± 10.23 in B16F10 cells and 42.91 ± 9.54 in MCF-7 cells, the CL-APN remarkably inhibited cell migration compared to any other group in both the cell lines. The results of the cell migration assay were consistent with the cytotoxicity assay results and CL-APN could be potentially used in the treatment of multiple tumor malignancies.	[174]
Folate (FA) and CUR	NLCs	FA-CUR-NLCs formulations exhibited small particle size of 127 nm. The IC_50_ (μmol/l) value of FA-CUR-NLCs (1.75 ± 0.29) was over 3.5-fold over CUR-NLCs (6.16 ± 0.67) in reducing viability of breast cancer cells, accounting for the highest anti-tumor activity. The effect of FA targeting and delivery ability of this NLCs formulation might be an effective tumor therapy strategy for treatment in breast carcinoma.	[150]
Kaempferol (KAE) and paclitaxel	NLCs	Particle size of 80 ± 3 nm for Kaempferol formulated into NLCs. The IC_50_ values for KAE and paclitaxel determined 44 ± 0.52 μM and 1.75 ± 0.36 nM, respectively. The moderated cell proliferation from 56 ± 26.8% to 44 ± 3.9% (*p*< 0.05) was demonstrated by KAE loaded NLCs. Paclitaxel cytotoxicity against MDA-MB 468 mice Breast cancer cells was increased by KAE-loaded NLCs. Synergistic anticancer activity was achieved by blocking apoptotic signaling and suppressing cancer cell cycle arrest in Sub G1 arrest, as well as down regulating anti-apoptotic Bcl-2 family gene levels.	[175]
β-lapachone plus DOX (LDNLC)	NLCs	The size distribution of LDNLC was approximately 100 nm with narrow distribution range, which was reflected by its relatively small PDI of 0.123 (<0.3). Compared to DOX mono-delivery NLC, in vitro cell studies in MCF-7 ADR cells revealed enhanced DOX retention (DNLC). In vivo anti-cancer studies on MCF-7 ADR tumor-bearing mice also demonstrated that LDNLC was substantially more effective than mono-delivery NLCs (DNLC and LNLC). The IC_50_ for MCF-7 ADR cells for LDNLC was calculated as 7.26 μM with a combination index of 0.31, thus indicating a remarkable synergism in the drug-resistant strain and could be a potential platform for overcoming MDR.	[176]
Quercetin and DOX	Biotin receptor-targeting nanoparticles (BNDQ)	Particle size of BNDQ were found to be 105.8 ± 1.4 nm and DOX tolerance in MCF-7/ADR cancer cells is reduced in vitro and in vivo. Quercetin decreased both the activity and the expression of P-gp in MCF-7/ADR cells could be a potential platform for overcoming MDR and It was found that the IC_50_ of BNDQ was 0.17 μg/mL significantly enhanced by the presence of free biotin compared to that in the absence of biotin in both MCF-7 and MCF-7/ADR cells.	[177]

## Data Availability

Not applicable.
